# Political violence, racial violence, and new gun ownership: results from the 2023 National Survey of Gun Policy

**DOI:** 10.1186/s40621-024-00527-z

**Published:** 2024-09-06

**Authors:** Rebecca Valek, Julie A. Ward, Vanya Jones, Cassandra K. Crifasi

**Affiliations:** 1 Department of Health Policy and Management, Center for Gun Violence Solutions, Johns Hopkins Bloomberg School of Public Health, Baltimore, MD USA; 2 School of Public Health, Oregon Health and Science University-Portland State University, Portland, OR USA; 3 Department of Medicine, Health, and Society, Vanderbilt College of Arts and Science, Vanderbilt University, Nashville, TN USA; 4 Department of Health, Behavior and Society, Johns Hopkins Bloomberg School of Public Health, Baltimore, MD USA

**Keywords:** Gun ownership, New gun owners, Purchasing motivations, Political violence, Racial violence

## Abstract

**Background:**

U.S. firearm sales surged during the COVID-19 pandemic, with many purchases by first-time firearm owners. The 2023 National Survey of Gun Policy sought to understand the public health implications of this surge by comparing the purchasing motivations and firearm policy views of pandemic-era first-time purchasers to prior gun owners.

**Methods:**

We fielded a nationally representative public opinion survey of U.S. adults (n = 3096) from 1/4/23 to 2/6/23. We oversampled for gun owners and Black, Hispanic, and Asian Americans. Survey weights were applied to generate representative estimates. New gun owners were identified through affirmative responses to: “Have you bought any guns since January 1, 2020?” and “Did you buy your first gun after January 1, 2020?” Recent purchasers were additionally asked whether concerns of 1) political or 2) racial violence motivated their purchase. Purchase motivations and gun policy support were examined among new and prior gun owners (n = 1002) and compared using logistic regression and predictive probabilities.

**Results:**

Overall, 11% of respondents reported purchasing a gun since 1/1/20, 35% for the first time. Among recent purchasers, larger proportions of Democrat, Black, Asian, and Hispanic respondents were new gun owners than Republican or white respondents. Compared to prior owners, odds were 4.5-times higher that new gun owners’ recent purchase was motivated by racial violence and 3.2-times higher for political violence.

Majority support was found for protective gun policies, with few differences by purchase recency or motivations. The only policy for which support by new and prior gun owners differed significantly was the permit-to-purchase policy (76% v. 63%, respectively). Similarly, few significant differences in support were observed when stratifying by purchase motivation. Notably, both those who reported recent purchase motivations of racial violence and of political violence expressed significantly higher support for a “stand-your-ground” policy compared to those who did not report such motivations.

**Conclusions:**

Racial and political violence appear to be larger concerns among new gun owners, motivating purchasing among demographic groups with traditionally lower gun ownership rates. These findings suggest a need for safety assurances amid racial and political tensions and growing gun ownership. Gun owners’ support for such policies remains strong.

**Supplementary Information:**

The online version contains supplementary material available at 10.1186/s40621-024-00527-z.

## Background

Firearm sales surged across the U.S. during the COVID-19 pandemic, with estimates of over four million excess firearms purchased from March to July 2020 (Schleimer et al. 2021). In this time period, an estimated 34% of all purchasers were new gun owners (Crifasi et al. 2021a). In addition to increasing individual gun ownership rates, these purchases increased household gun ownership rates, exposing over 11 million additional individuals to household firearm ownership (Miller et al. 2022). While little is known about the motivations behind this surge in purchasing, one study conducted in the summer of 2020 found that those reporting an intention to purchase a firearm over the next year had lower tolerance of uncertainty, stronger pandemic-related fears, and exaggerated threat expectancies compared to those who did not intend to purchase a firearm in the next year (Anestis and Bryan 2021). Another survey conducted between March 2020 and October 2021 reported that those who purchased a firearm during the pandemic expressed higher levels of agreement with various political beliefs (e.g., QAnon beliefs, pro-gun attitudes, Christian nationalism, COVID-19 skepticism) and exhibited higher levels of different mental health (e.g., suicidality, depression, alcohol use problems) and personality (e.g., desire for power, belief in a dangerous world) characteristics compared to both prior gun owners and non-gun owners (Hicks et al. 2023). Additionally, an examination of reasons for gun ownership among new and prior gun owners found that new gun owners more commonly cited multiple reasons for gun ownership as important or highly important, including reasons related to protection in ideological conflict, compared to prior gun owners (Ward et al. 2024).

This surge in firearm purchasing and new gun ownership occurred at the same time the nation saw reports of record-high rates of gun violence (Neuman 2023; Davis et al. 2023). The Centers for Disease Control and Prevention’s (CDC) Underlying Cause of Death database reported record-high numbers of gun deaths in 2020 and 2021 (Davis et al. 2023; Centers for Disease Control and Prevention 2024). Between 2019 and 2021, the national gun homicide rate increased by 45%, the largest two-year increase that the CDC has reported to date, and the gun suicide rate increased by 10% (Davis et al. 2023). An analysis of national gun violence rates using police reports of gun-related injuries and deaths documented in the Gun Violence Archive reported similar increases, with a 34% increase in nonfatal firearm injury and a 28% increase in firearm-related deaths during the pandemic compared to prior years (Sun et al. 2022). The U.S. also saw an increase in the number of hate crimes, especially those targeting Black and Asian Americans (U.S. Department of Justice 2023). Simultaneously, the COVID-19 pandemic and co-occurring social justice and police reform-related protests may have spurred uncertainty about economic wellbeing, status, or stability of established hierarchies and norms (Anestis et al. 2023). Such concerns have been associated with symbolically protective gun acquisition (Anestis et al. 2023; Warner and Steidley 2021). In short, increased concern about general crime, group-specific safety, or political and social unrest may uniquely influence firearm-related beliefs and behaviors (Anestis et al. 2023; Barnes and Ephross 1994).

Given that gun ownership has been associated with increased risks of firearm homicide, suicide, and unintentional shootings, there is a need to further characterize and understand the recent gun purchasing surge, particularly within the context of persistently heightened social and political tensions (Siegel et al. 2013; Miller et al. 2002; Wiebe 2003). Despite our knowledge of the surge in gun purchases during the pandemic, less is known about the motivations behind these recent purchasing behaviors and potential differences between new and prior gun owners or among different racial and ethnic and political groups. This information is critical to understanding how to reduce violence and promote safety through strategies such as education campaigns or policy change. This study sought to examine purchasing behaviors and motivations as well as support for policies and programs to reduce gun violence, comparing prior gun owners to those who bought guns for the first time during the COVID-19 pandemic.

## Methods

We fielded the 2023 National Survey of Gun Policy using NORC at the University of Chicago’s AmeriSpeak panel from January 4 to February 6, 2023 (n = 3096) to examine public opinion on gun policy. The AmeriSpeak Panel is drawn from the NORC National Frame, a nationally representative, probability-based panel of adults ages 18 and older that uses address-based sampling to cover 97% of U.S. households (NORC at the University of Chicago 2022). Interviews were administered online and by phone in both English and Spanish. All question blocks were asked in random order to protect against potential for systematic priming.

The survey completion rate was 76.5%. We oversampled for gun owners (n = 1002) and Black Americans, Hispanic Americans, and Asian Americans. This oversampling allowed for an assessment of different purchasing behaviors and motivations among various demographic groups, particularly those groups for which there were increases in gun ownership during the pandemic. Race and ethnicity were self-reported by the survey participants and participants were classified into one of five mutually exclusive ethnoracial categories: non-Hispanic white, non-Hispanic Black, non-Hispanic Asian, non-Hispanic other race or multiracial, or Hispanic of any race.

Gun ownership was determined through two questions: “Do you happen to have in your home or garage any guns or revolvers?” and “Do any of these guns personally belong to you?” A gun owner was defined as a respondent who was the personal owner of at least one firearm. Recent gun ownership and purchasing were identified through the questions: “Have you bought any guns since January 1, 2020?” and “Did you buy your first gun after January 1, 2020?” Those who purchased any guns since January 1, 2020, were labelled “recent purchasers” and recent purchasers who purchased their first gun since January 1, 2020, were labelled “new gun owners.” Gun owners whose first purchase was before January 1, 2020, were referred to as “prior gun owners.” Recent purchasers were asked about the motivations for their recent purchase: “Was your purchase motivated by concerns of racial violence?” and “Was your purchase motivated by concerns of political violence?” Eleven additional questions regarding reasons for gun ownership, including more conventional reasons like hunting and protection at home, were asked of all gun owners. The results of these questions were previously published (Ward et al. 2024).

We used detailed information about respondents’ demographic characteristics, including sex, age, race and ethnicity, education, income, employment status, region of residence, and political party affiliation provided by NORC. Supplemental Table 1 in Additional File 1 compares the survey weighted and unweighted sociodemographic characteristics and political party affiliation of the study sample in 2023 to national data (U.S. Census Bureau 2024a, b; Bureau of Labor Statistics 2024; American National Election Studies 2022).

We examined respondents’ support for 42 gun-related policies. These policies were grouped into ten categories: license and background check policies, prohibited persons policies, assault weapon and ammunition policies, policies affecting gun dealers, temporary firearm removal policies, policies on carrying guns in public, policies prohibiting a person convicted of various crimes from having a gun for 10 years, funding-related policies, safe storage policies, and other policies. Support was measured via a 5-point Likert scale ranging from ‘strongly favor’ to ‘strongly oppose’. We created a dichotomous support measure comparing ‘somewhat favor’ and ‘strongly favor’ to the other options to indicate the proportion in support of each policy.

Logistic regression was used to compare differences in gun ownership and purchase motivation among demographic subgroups (e.g., racial and ethnic group, political affiliation) and to compare differences in unadjusted policy support by gun ownership and purchase motivation. We conducted analyses using survey weights provided by NORC to adjust for known sampling deviations and survey nonresponse and to ensure the sample was representative of the U.S. population. Results are presented as weighted proportions with 95% confidence intervals. Average predicted probabilities of policy support were calculated as a sensitivity analysis to assess whether observed differences remained after accounting for demographic variables: ethnoracial group, political party identification, sex, age, income, and living in a metropolitan area. All analyses were conducted using the *svy* command in Stata version 17.0. This study was reviewed and approved by the Johns Hopkins Institutional Review Board.

## Results

Overall, 11% (95% CI: 9.4–12.4) of survey respondents reported having bought any guns since January 1, 2020, 35% of whom were new gun owners (95% CI: 28.4–42.0). A significantly larger proportion of Republicans reported having made a recent firearm purchase compared to Democrats (Republicans: 15%, 95% CI: 12.4–18.8; Democrats: 6%, 95% CI: 4.0–7.3). When stratifying by race and ethnicity, a significant difference in proportions of having made a recent firearm purchase only existed for one racial group: people who identified as non-Hispanic Asian. A significantly lower proportion of Asian respondents reported having made a recent firearm purchase (5%; 95% CI: 2.8–8.6) compared to white (11%; 95% CI: 8.9–12.7), Black (11%; 95% CI: 8.2–14.7), other/multiracial (16%; 95% CI: 7.9–30.3), or Hispanic (13%; 95% CI: 9.4–17.5) respondents. Among those who made recent purchases, proportions of new gun ownership were significantly higher among respondents identifying as Democrat (51%; 95% CI: 35.6–66.0), Black (53%; 95% CI: 37.1–67.5), Asian (70%; 95% CI: 42.4–88.5), or Hispanic (55%; 95% CI: 38.3–71.0) compared to those identifying as Republican (23%; 95% CI: 15.2–34.0) or white (24%; 95% CI: 16.8–32.6). Table 1 describes patterns in firearm purchasing and motivations during the COVID-19 pandemic by political party and by race and ethnicity.

**Table 1:** Gun purchasing behaviors during COVID-19 by political party and race and ethnicity

	Overall % (CI) (N = 3096)	Political party			Race and ethnicity				
		Democrat % (CI) (N = 1199)	Independent % (CI) (N = 1163)	Republican % (CI) (N = 730)	White, non-Hispanic % (CI) (N = 1391)	Black, non-Hispanic % (CI) (N = 668)	Asian, non-Hispanic % (CI) (N = 338)	Other/multi, non-Hispanic % (CI) (N = 64)	Hispanic % (CI) (N = 635)
Have you bought any guns since January 1, 2020?	10.8 (9.4, 12.4)	5.5 (4.0, 7.3)	12.2*** (9.8, 15.1)	15.3*** (12.4, 18.8)	10.7 (8.9, 12.7)	11.1 (8.2, 14.7)	5.0**^ (2.8, 8.6)	**16.2+ + **(7.9, 30.3)	**12.9+ + **(9.4, 17.5)
Among those who bought guns since January 1, 2020^1^									
Did you buy your first gun after January 1, 2020?	34.9 (28.4, 42.0)	50.9 (35.6, 66.0)	39.5 (28.8, 51.2)	23.3**^ (15.2, 34.0)	23.8 (16.8, 32.6)	52.5*** (37.1, 67.5)	70.4*** (42.4, 88.5)	**18.6+ **(2.7, 65.3)	55.2*** (38.3, 71.0)
Was your purchase motivated by concerns of racial violence?	17.1 (12.8, 22.5)	22.9 (13.3, 36.5)	16.9 (10.4, 26.3)	14.9 (8.9, 23.8)	10.5 (6.4, 16.7)	35.6*** (22.4, 51.4)	41.5** (16.2, 72.4)	18.6 (2.7, 65.3)	22.2 (11.2, 39.1)
Was your purchase motivated by concerns of political violence?	24.3 (18.7, 31.0)	34.1 (20.4, 51.0)	27.3 (18.1, 38.9)	17.0* (10.5, 26.1)	19.2 (12.9, 27.5)	27.0 (15.8, 42.3)	40.8 (15.6, 72.1)	35.0 (9.1, 74.4)	34.1 (19.2, 53.0)

Statistically significant difference indicated in bold

^*^p ≤ 0.05, **p ≤ 0.01, ***p ≤ 0.001 for comparisons to Democrat or to white, non-Hispanic

^*p* ≤ 0.05, ^^*p* ≤ 0.01, ^^^*p* ≤ 0.001 for comparing Republican to Independent or comparisons to Black, non-Hispanic

+ *p* ≤ 0.05, + + *p* ≤ 0.01, + + + *p* ≤ 0.001 for comparisons to Asian, non-Hispanic

^1^These questions were only asked of those who bought guns since January 1, 2020. Overall N = 327, Democrat N = 74, Independent N = 131, Republican N = 122, White, non-Hispanic N = 163, Black, non-Hispanic N = 67, Asian, non-Hispanic N = 19, Other/Multi, non-Hispanic N = 11, Hispanic N = 67

### Purchase motivations

Overall, 17% (95% CI: 12.8–22.5) of recent firearm purchasers reported that their purchase was motivated by concerns of racial violence and 24% (95% CI: 18.7–31.0) reported that their purchase was motivated by concerns of political violence (Table 1). New gun owners reported significantly different purchase motivations compared to recent purchases among prior gun owners. When controlling for political party and various demographic characteristics, odds of reporting recent purchases motivated by concerns of racial violence and concerns of political violence were 4.5 (95% CI: 2.0–10.1) and 3.2 (95% CI: 1.5–6.9) times greater, respectively, among new gun owners compared to prior gun owners who also made a recent purchase (Table 2). Among recent purchasers, significantly larger proportions of Black (36%; 95% CI: 22.4–51.4) and Asian (42%; 95% CI: 16.2–72.4) respondents reported recent purchases motivated by concerns of racial violence than white respondents (11%; 95% CI: 6.4–16.7; Table 1). A significantly higher proportion of recent purchasers identifying as Democrats reported being motivated by political violence concerns (34%; 95% CI: 20.4–51.0) compared to Republican recent purchasers (17%; 95% CI: 10.5–26.1; Table 1).

**Table 2:** Adjusted odds of gun purchasing behaviors and motivations during COVID-19

	Recent Purchasing^1^ OR (95% CI)	New Gun Ownership^2,3^ OR (95% CI)	Purchase motivated by racial violence concerns^2^ OR (95% CI)	Purchase motivated by political violence concerns^2^ OR (95% CI)
New gun ownership^3^				
Prior gun owner (ref)	–	–	–	–
New gun owner	–	–	**4.5 (2.0, 10.1)**	**3.2 (1.5, 6.9)**
Political party				
Democrat (ref)	–	–	–	–
Independent	**2.5 (1.6, 3.7)**	0.8 (0.3, 2.0)	1.0 (0.3, 2.7)	0.7 (0.3, 1.8)
Republican	**3.3 (2.2, 5.1)**	0.4 (0.2, 1.3)	1.4 (0.5, 4.4)	**0.3 (0.1, 0.8)**
Race and ethnicity				
White, non-Hispanic (ref)	–	–	–	–
Black, non-Hispanic	**2.0 (1.2, 3.1)**	2.6 (1.0, 6.8)	**4.1 (1.3, 12.6)**	0.9 (0.3, 2.5)
Asian, non-Hispanic	**0.5 (0.3, 0.9)**	**12.6 (3.2, 50.1)**	4.1 (1.0, 17.0)	1.5 (0.4, 6.2)
Other/multi, non-Hispanic	1.5 (0.6, 3.8)	0.8 (0.1, 5.9)	4.0 (0.8, 19.8)	3.3 (0.8, 12.8)
Hispanic	**1.5 (1.0, 2.4)**	**3.7 (1.6, 8.5)**	2.6 (0.9, 7.4)	1.4 (0.5, 3.9)
Sex				
Male (ref)	–	–	–	–
Female	**0.5 (0.3, 0.7)**	1.2 (0.6, 2.5)	0.9 (0.4, 2.0)	0.5 (0.2, 1.3)
Age				
35–64 years old (ref)	–	–	–	–
18–34 years old	0.9 (0.6, 1.3)	1.1 (0.5, 2.2)	0.5 (0.2, 1.4)	0.5 (0.2, 1.3)
65 + years old	**0.5 (0.4, 0.8)**	0.7 (0.3, 1.8)	1.9 (0.7, 5.2)	1.2 (0.5, 3.1)
Income				
$75,000 + (ref)	–	–	–	–
$35,000–$74,999	0.7 (0.5, 1.0)	**2.6 (1.2, 5.4)**	0.8 (0.3, 2.1)	0.5 (0.2, 1.2)
< $35,000	**0.5 (0.3, 0.7)**	1.0 (0.4, 2.3)	**3.0 (1.1, 8.8)**	1.2 (0.4, 3.6)
Urbanicity				
Metro area resident (ref)	–	–	–	–
Non-metro area resident	**1.6 (1.1, 2.4)**	1.3 (0.7, 2.8)	1.7 (0.7, 4.2)	1.3 (0.5, 3.2)

Statistically significant differences indicated in bold

*OR* odds ratio. *CI* confidence interval

^1^Recent purchasing is defined as those who reported purchasing any guns since January 1, 2020

^2^These questions were only asked of those who bought guns since January 1, 2020

^3^New gun ownership is defined as those who purchased their first gun since January 1, 2020. New gun ownership is only included as a variable in the models for the purchase motivation questions

### Policy support

Support for the gun-related policies was generally high among both gun owners and non-gun owners (Supplemental Table 2 in Additional File 1) and among new and prior gun owners (Table 3) for most protective policies; few significant differences in policy support were observed. Support only differed significantly between new and prior gun owners for one policy: firearm purchaser licensing/permit-to-purchase. New gun owners reported significantly higher support for a policy requiring a person to obtain a license from a local law enforcement agency before buying a gun to verify their identity and ensure that they are not legally prohibited from having a gun (76%; 95% CI: 64.7–84.6) compared to prior gun owners (63%; 95% CI: 59.2–67.1).

**Table 3:** Support for 42 different gun policies by new versus prior gun ownership

	Overall gun owner % (CI) (N = 1002)	Prior gun owner % (CI) (N = 904)	New gun owner % (CI) (N = 119)
License and background check policies			
Requiring a background check system for all gun sales to make sure a purchaser is not legally prohibited from having a gun	84.1 (81.0, 86.8)	83.6 (80.3, 86.5)	87.2 (78.0, 93.0)
Requiring a person to obtain a license from a local law enforcement agency before buying a gun to verify their identity and ensure that they are not legally prohibited from having a gun	64.4 (60.5, 68.0)	63.2 (59.2, 67.1)	76.0* (64.7, 84.6)
Requiring a license to buy a gun if you could substitute a valid concealed carry license^1^	20.9 (16.4, 26.3)	19.4 (14.9, 24.9)	36.3 (18.0, 59.7)
Requiring that a person be fingerprinted for the background check to verify a person’s identity and link it to any relevant criminal records	71.2 (67.4, 74.6)	70.1 (66.2, 73.8)	80.8 (69.1, 88.7)
Extending the time to conduct a background check to up to 10 days	62.6 (58.7, 66.3)	62.5 (58.5, 66.4)	64.4 (51.9, 75.3)
Prohibiting the sale of a gun before a background check is complete	65.1 (61.3, 68.8)	65.0 (60.9, 68.8)	63.0 (50.9, 73.7)
Prohibited persons policies			
Prohibiting a person subject to a temporary domestic violence restraining order from having a gun for the duration of the order	79.2 (75.8, 82.3)	78.9 (75.3, 82.2)	80.2 (69.9, 87.7)
Extending domestic violence-related gun prohibitions to include couples who have dated	57.1 (53.2, 61.0)	57.3 (53.2, 61.3)	55.1 (42.9, 66.8)
Prohibiting a person convicted of a serious crime as a juvenile from having a gun for 10 years	77.8 (74.5, 80.8)	77.8 (74.3, 80.9)	77.3 (67.1, 85.1)
Prohibiting a person under the age of 21 from having a handgun	57.7 (53.7, 61.5)	56.8 (52.7, 60.8)	68.6 (56.6, 78.5)
Prohibiting a person convicted of two or more misdemeanor crimes involving illegal drugs in a five-year period from having a gun for five years	61.0 (57.1, 64.8)	61.1 (57.1, 65.1)	62.1 (50.2, 72.7)
Prohibiting a person convicted of two or more DWI or DUIs in a five-year period from having a gun for five years	52.7 (48.8, 56.6)	52.2 (48.1, 56.3)	60.9 (48.7, 71.8)
Assault weapon and ammunition policies			
Banning the sale of military-style, semi-automatic assault weapons that are capable of shooting more than 10 rounds of ammunition without reloading	44.2 (40.4, 48.2)	44.6 (40.5, 48.7)	41.1 (29.8, 53.4)
Banning the sale of large-capacity ammunition clips or magazines that allow some guns to shoot more than 10 bullets before reloading	43.4 (39.5, 47.3)	43.8 (39.8, 48.0)	39.3 (28.1, 51.7)
Requiring an owner of a semi-automatic rifle, that ejects and rechambers a new round after each shot allowing a person to fire the rifle as quickly as the trigger can be pulled, to be at least 21 years of age	65.9 (62.1, 69.4)	65.5 (61.6, 69.3)	70.9 (59.0, 80.4)
Policies affecting gun dealers			
Allowing the Bureau of Alcohol, Tobacco and Firearms to temporarily take away a gun dealer’s license if an audit reveals record-keeping violations and the dealer cannot account for 20 or more of his guns	80.2 (76.7, 83.2)	80.7 (77.1, 83.9)	74.6 (62.4, 83.9)
Allowing cities to sue licensed gun dealers when there is strong evidence that the gun dealer’s careless sales practices allowed many criminals to obtain guns	68.6 (64.9, 72.2)	68.5 (64.5, 72.2)	67.8 (56.1, 77.7)
Allowing the information about which gun dealers sell the most guns used in crimes to be available to the police and the public so that those gun dealers can be prioritized for greater oversight	61.1 (57.2, 64.9)	61.1 (57.0, 65.1)	58.4 (46.3, 69.6)
Temporary firearm removal policies			
Allowing family members to ask the court to temporarily remove guns from a relative who they believe is at risk of harming himself or others	72.3 (68.6, 75.7)	72.8 (68.9, 76.3)	65.2 (53.1, 75.6)
Authorizing law enforcement officers to temporarily remove guns from individuals who the officer determines pose an immediate threat of harm to self or others	65.5 (61.7, 69.2)	66.0 (61.9, 69.8)	59.5 (47.3, 70.7)
Allowing licensed healthcare providers to ask the court to temporarily remove guns from a patient who they believe is at risk of harming himself or others	71.6 (67.8, 75.0)	71.4 (67.5, 75.1)	70.1 (58.5, 79.6)
Policies on carrying guns in public			
Requiring a person who has applied for a license to carry a concealed gun in public to pass a test demonstrating that they can safely and lawfully handle a gun in common situations they might encounter	68.3 (64.5, 71.9)	67.9 (63.8, 71.7)	71.6 (59.3, 81.4)
Allowing a person who can legally carry a concealed gun to bring that gun onto a college or university campus	41.8 (38.0, 45.8)	41.7 (37.6, 45.8)	38.1 (26.9, 50.8)
Allowing a person who can legally carry a concealed gun to bring that gun onto school grounds for kindergarten through 12th grade	36.3 (32.6, 40.2)	35.9 (32.1, 40.0)	40.2 (29.1, 52.5)
Allowing a person who can legally own a gun to carry a loaded, concealed handgun in public without having to obtain a concealed carry license	34.5 (30.9, 38.2)	33.9 (30.1, 37.8)	39.0 (28.1, 51.2)
Requiring a state to recognize a concealed carry permit from another state, even if that other state’s firearm concealed carry permitting standards are lower	62.7 (58.7, 66.4)	61.7 (57.6, 65.7)	68.3 (56.4, 78.2)
Prohibiting the open carrying of a gun (i.e., carrying in a manner that makes it visible) at a public demonstration or rally	48.0 (44.1, 51.9)	47.4 (43.3, 51.6)	53.7 (41.6, 65.3)
Prohibiting a person from bringing a gun into a government building	59.4 (55.5, 63.2)	58.7 (54.5, 62.7)	69.3 (57.4, 79.1)
Policies prohibiting a person convicted of each of these crimes from having a gun for 10 years			
Public display of a gun in a threatening manner, excluding self-defense	69.8 (66.0, 73.3)	70.0 (66.1, 73.7)	69.3 (57.5, 79.0)
Assault and battery that does not result in serious injury or involve a lethal weapon	49.8 (45.9, 53.7)	50.1 (46.0, 54.2)	51.1 (39.0, 63.0)
Carrying a concealed gun without a permit	46.4 (42.5, 50.3)	46.2 (42.1, 50.3)	49.8 (37.8, 61.9)
Drunk and disorderly conduct	37.7 (34.0, 41.6)	37.1 (33.3, 41.2)	46.2 (34.3, 58.6)
Funding-related policies			
Directing federal government funding to states that want to establish licensing systems for handgun purchasers	49.8 (45.8, 53.7)	49.9 (45.8, 54.0)	47.4 (35.6, 59.6)
Funding community-based gun violence prevention programs that provide outreach, conflict mediation, and social support for individuals at high risk of gun violence	61.8 (57.8, 65.6)	61.5 (57.3, 65.5)	64.6 (52.3, 75.2)
Directing public funding to dispatching a clinician to accompany police officers on calls involving individuals displaying symptoms of mental illness	62.6 (58.7, 66.4)	62.1 (58.0, 66.1)	64.1 (52.0, 74.7)
Directing public funding for community-based mental health programs to respond to calls involving individuals displaying symptoms of mental illness	65.8 (61.9, 69.5)	65.1 (61.0, 69.0)	69.0 (56.8, 79.0)
Redirecting government funding currently spend on the police to social services for people at risk of gun violence	29.3 (25.9, 33.0)	28.8 (25.3, 32.5)	31.9 (21.5, 44.4)
Funding, through public insurance, hospital-based gun violence prevention programs that offer counseling to address psychological trauma	54.0 (50.1, 58.0)	53.4 (49.3, 57.5)	54.8 (42.6, 66.4)
Safe storage policies			
Requiring first-time gun purchasers to take a safety course on safe handling and storage before buying a gun	78.8 (75.4, 81.8)	78.3 (74.7, 81.5)	80.5 (69.5, 88.2)
Requiring by law that a person lock up the guns in their home when not in use to prevent handling by children or teenagers without adult supervision	58.0 (54.1, 61.9)	56.8 (52.7, 60.9)	67.4 (55.1, 77.8)
Other policies			
Allowing a person with a gun who feels a threat of serious injury from another person to shoot or kill that threatening person, even if the gun owner could safely retreat	35.9 (32.2, 39.7)	35.5 (31.7, 39.5)	35.9 (25.1, 48.3)
Prohibiting the possession of guns that do not have serial numbers	66.2 (62.3, 69.8)	66.0 (61.9, 69.8)	68.0 (55.6, 78.3)

Statistically significant difference indicated in bold

^*^p ≤ 0.05, **p ≤ 0.01, *** *p* ≤ 0.001

^1^This question was only asked of those opposed to requiring a license from a local law enforcement agency before buying a gun to verify their identity and ensure that they are not legally prohibited from having a gun. Overall Gun Owner N = 352, Prior Gun Owner N = 325, New Gun Owner N = 32

Similarly, few significant differences in support for the 42 policies of interest were observed when stratifying by whether recent purchases were motivated by concerns of racial or political violence (Table 4). Significantly larger proportions of those whose recent purchases were motivated by concerns of racial violence expressed support for a prohibition on handgun ownership for those under the age of 21 (67%; 95% CI: 51.6–78.9) compared to those whose recent purchases were not motivated by concerns of racial violence (50%; 95% CI: 41.8–57.8). Significantly larger proportions of those whose recent purchases were motivated by concerns of political violence expressed support for child access prevention laws that require a person lock up their guns when not in use to prevent handling by children or teenagers without adult supervision (66%; 95% CI: 51.6–78.4) compared to those whose recent purchases were not motivated by concerns of political violence (49%; 95% CI: 40.4–56.7). Recent political-violence-motivated purchasers also reported significantly higher proportions of support for requiring concealed carry applicants to pass a test demonstrating that they can safely and lawfully handle a gun in common situations they may encounter (79%; 95% CI: 66.8–87.2) relative to those whose recent purchases were not motivated by concerns of political violence (60%, 95% CI: 52.0–68.1). Three laws had significant differences in support when stratifying by either motivation, with higher support among those reporting concerns of either type of violence: requiring a license to buy a gun if you could substitute a valid concealed carry license; redirecting government funding currently spent on the police to social services for people at risk of gun violence; and the stand-your-ground law, which allows a person with a gun who feels a threat of serious injury from another person to shoot or kill that threatening person, even if the gun owner could safely retreat.

**Table 4:** Support for 42 different gun policies by motivation for recent gun purchases

	All recent purchasers % (CI) (N = 327)	Recent purchase motivated by concerns of racial violence^1^		Recent purchase motivated by concerns of political violence^1^	
		Yes % (CI) (N = 69)	No % (CI) (N = 258)	Yes % (CI) (N = 83)	No % (CI) (N = 244)
License and background check policies					
Requiring a background check system for all gun sales to make sure a purchaser is not legally prohibited from having a gun	79.3 (73.1, 84.4)	82.4 (69.2, 90.7)	78.7 (71.5, 84.4)	80.7 (67.4, 89.4)	78.9 (71.6, 84.7)
Requiring a person to obtain a license from a local law enforcement agency before buying a gun to verify their identity and ensure that they are not legally prohibited from having a gun	55.7 (48.4, 62.7)	66.9 (51.7, 79.3)	53.4 (45.3, 61.4)	63.0 (48.4, 75.6)	53.4 (45.1, 61.5)
Requiring a license to buy a gun if you could substitute a valid concealed carry license^2^	17.5 (11.7, 25.5)	41.6 (19.4, 67.8)	14.1* (8.7, 22.0)	34.3 (17.3, 56.5)	13.4* (8.0, 21.7)
Requiring that a person be fingerprinted for the background check to verify a person’s identity and link it to any relevant criminal records	61.8 (54.6, 68.5)	68.5 (52.5, 81.0)	60.5 (52.3, 68.0)	69.1 (54.4, 80.8)	59.5 (51.1, 67.3)
Extending the time to conduct a background check to up to 10 days	52.6 (45.4, 59.7)	57.4 (41.7, 71.7)	51.6 (43.5, 59.6)	58.2 (43.2, 71.8)	50.8 (42.6, 58.9)
Prohibiting the sale of a gun before a background check is complete	58.7 (51.6, 65.5)	58.7 (43.3, 72.6)	58.7 (50.7, 66.4)	65.6 (50.9, 77.7)	56.5 (48.3, 64.5)
Prohibited persons policies					
Prohibiting a person subject to a temporary domestic violence restraining order from having a gun for the duration of the order	73.1 (66.4, 78.9)	73.4 (58.7, 84.3)	73.1 (65.4, 79.6)	71.8 (57.9, 82.6)	73.5 (65.6, 80.2)
Extending domestic violence-related gun prohibitions to include couples who have dated	50.7 (43.5, 57.9)	48.4 (33.5, 63.5)	51.2 (43.1, 59.2)	46.4 (32.2, 61.3)	52.1 (43.8, 60.2)
Prohibiting a person convicted of a serious crime as a juvenile from having a gun for 10 years	74.2 (67.9, 79.7)	74.9 (60.6, 85.3)	74.1 (66.8, 80.2)	72.0 (58.0, 82.8)	74.9 (67.5, 81.0)
Prohibiting a person under the age of 21 from having a handgun	52.7 (45.5, 59.7)	66.6 (51.6, 78.9)	49.8* (41.8, 57.8)	52.3 (37.6, 66.6)	52.8 (44.6, 60.9)
Prohibiting a person convicted of two or more misdemeanor crimes involving illegal drugs in a five-year period from having a gun for five years	53.8 (46.7, 60.9)	65.0 (49.7, 77.7)	51.5 (43.5, 59.5)	61.6 (47.0, 74.4)	51.3 (43.1, 59.5)
Prohibiting a person convicted of two or more DWI or DUIs in a five-year period from having a gun for five years	50.0 (42.9, 57.2)	59.9 (44.5, 73.6)	48.0 (40.0, 56.1)	61.8 (47.1, 74.7)	46.2 (38.1, 54.5)
Assault weapon and ammunition policies					
Banning the sale of military-style, semi-automatic assault weapons that are capable of shooting more than 10 rounds of ammunition without reloading	26.9 (21.0, 33.8)	33.8 (21.2, 49.3)	25.5 (19.0, 33.3)	35.9 (23.1, 51.1)	24.0 (17.7, 31.8)
Banning the sale of large-capacity ammunition clips or magazines that allow some guns to shoot more than 10 bullets before reloading	25.7 (20.0, 32.5)	36.1 (23.0, 51.6)	23.6 (17.4, 31.2)	30.2 (19.1, 44.1)	24.3 (17.7, 32.3)
Requiring an owner of a semi-automatic rifle, that ejects and rechambers a new round after each shot allowing a person to fire the rifle as quickly as the trigger can be pulled, to be at least 21 years of age	57.9 (50.8, 64.7)	70.3 (54.5, 82.4)	55.3 (47.3, 63.1)	66.5 (51.5, 78.8)	55.1 (46.9, 63.1)
Policies affecting gun dealers					
Allowing the Bureau of Alcohol, Tobacco and Firearms to temporarily take away a gun dealer’s license if an audit reveals record-keeping violations and the dealer cannot account for 20 or more of his guns	73.6 (66.9, 79.4)	81.0 (66.7, 90.1)	72.1 (64.4, 78.7)	73.5 (58.2, 84.7)	73.6 (66.0, 80.1)
Allowing cities to sue licensed gun dealers when there is strong evidence that the gun dealer’s careless sales practices allowed many criminals to obtain guns	61.2 (54.1, 67.8)	65.8 (50.5, 78.4)	60.2 (52.2, 67.7)	66.1 (52.0, 77.9)	59.6 (51.4, 67.3)
Allowing the information about which gun dealers sell the most guns used in crimes to be available to the police and the public so that those gun dealers can be prioritized for greater oversight	47.3 (40.2, 54.5)	51.3 (36.2, 66.2)	46.4 (38.5, 54.6)	53.2 (38.6, 67.3)	45.4 (37.3, 53.6)
Temporary firearm removal policies					
Allowing family members to ask the court to temporarily remove guns from a relative who they believe is at risk of harming himself or others	62.4 (55.3, 69.0)	71.8 (57.0, 83.0)	60.5 (52.4, 68.0)	66.5 (51.6, 78.7)	61.1 (52.9, 68.7)
Authorizing law enforcement officers to temporarily remove guns from individuals who the officer determines pose an immediate threat of harm to self or others	49.8 (42.6, 56.9)	50.6 (35.6, 65.6)	49.6 (41.6, 57.6)	47.1 (32.9, 61.7)	50.6 (42.4, 58.8)
Allowing licensed healthcare providers to ask the court to temporarily remove guns from a patient who they believe is at risk of harming himself or others	63.4 (56.4, 69.9)	73.6 (58.9, 84.4)	61.3 (53.4, 68.7)	72.6 (58.7, 83.2)	60.5 (52.3, 68.1)
Policies on carrying guns in public					
Requiring a person who has applied for a license to carry a concealed gun in public to pass a test demonstrating that they can safely and lawfully handle a gun in common situations they might encounter	64.8 (57.8, 71.2)	74.6 (60.6, 84.9)	62.8 (54.8, 70.1)	78.7 (66.8, 87.2)	60.3* (52.0, 68.1)
Allowing a person who can legally carry a concealed gun to bring that gun onto a college or university campus	50.7 (43.5, 57.8)	41.1 (27.3, 56.4)	52.7 (44.6, 60.6)	60.1 (45.1, 73.3)	47.7 (39.6, 55.9)
Allowing a person who can legally carry a concealed gun to bring that gun onto school grounds for kindergarten through 12th grade	43.5 (36.6, 50.6)	39.9 (26.2, 55.4)	44.2 (36.4, 52.3)	44.3 (30.6, 59.0)	43.2 (35.3, 51.4)
Allowing a person who can legally own a gun to carry a loaded, concealed handgun in public without having to obtain a concealed carry license	42.2 (35.5, 49.2)	55.8 (40.5, 70.0)	39.4 (32.1, 47.2)	49.0 (34.7, 63.5)	40.0 (32.5, 48.0)
Requiring a state to recognize a concealed carry permit from another state, even if that other state’s firearm concealed carry permitting standards are lower	69.0 (61.7, 75.4)	80.4 (67.9, 88.8)	66.6 (58.3, 74.0)	76.3 (60.7, 87.0)	66.6 (58.2, 74.1)
Prohibiting the open carrying of a gun (i.e., carrying in a manner that makes it visible) at a public demonstration or rally	35.9 (29.2, 43.3)	48.1 (33.3, 63.3)	33.4 (25.9, 41.8)	45.2 (31.1, 60.2)	32.9 (25.4, 41.4)
Prohibiting a person from bringing a gun into a government building	52.7 (45.5, 59.7)	57.2 (42.0, 71.1)	51.7 (43.7, 59.7)	59.9 (45.3, 72.9)	50.3 (42.1, 58.5)
Policies prohibiting a person convicted of each of these crimes from having a gun for 10 years					
Public display of a gun in a threatening manner, excluding self-defense	67.4 (60.5, 73.6)	71.1 (56.0, 82.6)	66.7 (58.9, 73.7)	65.3 (50.6, 77.6)	68.1 (60.2, 75.1)
Assault and battery that does not result in serious injury or involve a lethal weapon	45.9 (38.9, 53.2)	52.8 (37.6, 67.6)	44.5 (36.7, 52.7)	47.6 (33.3, 62.2)	45.4 (37.4, 53.7)
Carrying a concealed gun without a permit	43.1 (36.1, 50.3)	48.4 (33.5, 63.5)	42.0 (34.1, 50.2)	49.1 (34.7, 63.6)	41.1 (33.2, 49.6)
Drunk and disorderly conduct	33.9 (27.3, 41.1)	41.0 (27.1, 56.4)	32.4 (25.2, 40.6)	38.5 (25.3, 53.8)	32.4 (25.1, 40.6)
Funding-related policies					
Directing federal government funding to states that want to establish licensing systems for handgun purchasers	41.6 (34.6, 48.9)	51.5 (36.1, 66.6)	39.5 (31.8, 47.8)	43.2 (29.3, 58.2)	41.0 (33.1, 49.4)
Funding community-based gun violence prevention programs that provide outreach, conflict mediation, and social support for individuals at high risk of gun violence	53.5 (46.2, 60.6)	56.1 (40.6, 70.5)	52.9 (44.8, 60.9)	53.5 (38.8, 67.7)	53.5 (45.1, 61.6)
Directing public funding to dispatching a clinician to accompany police officers on calls involving individuals displaying symptoms of mental illness	53.7 (46.5, 60.8)	58.2 (42.4, 72.4)	52.8 (44.6, 60.8)	65.6 (50.8, 78.0)	49.8 (41.6, 58.1)
Directing public funding for community-based mental health programs to respond to calls involving individuals displaying symptoms of mental illness	56.5 (49.2, 63.5)	69.0 (52.7, 81.6)	53.9 (45.8, 61.9)	65.5 (49.8, 78.3)	53.7 (45.3, 61.8)
Redirecting government funding currently spend on the police to social services for people at risk of gun violence	23.6 (18.1, 30.1)	40.4 (26.6, 56.0)	20.1** (14.3, 27.4)	34.0 (22.4, 47.9)	20.2* (14.1, 28.0)
Funding, through public insurance, hospital-based gun violence prevention programs that offer counseling to address psychological trauma	47.0 (39.9, 54.2)	57.0 (41.4, 71.3)	44.9 (37.0, 53.1)	52.3 (37.6, 66.5)	45.3 (37.2, 53.7)
Safe storage policies					
Requiring first-time gun purchasers to take a safety course on safe handling and storage before buying a gun	74.2 (67.6, 79.8)	75.7 (60.8, 86.2)	73.9 (66.3, 80.2)	75.2 (61.3, 85.3)	73.8 (66.1, 80.3)
Requiring by law that a person lock up the guns in their home when not in use to prevent handling by children or teenagers without adult supervision	52.8 (45.6, 59.9)	64.0 (48.2, 77.2)	50.4 (42.4, 58.5)	66.3 (51.6, 78.4)	48.5* (40.4, 56.7)
Other policies					
Allowing a person with a gun who feels a threat of serious injury from another person to shoot or kill that threatening person, even if the gun owner could safely retreat	39.9 (33.2, 46.9)	53.7 (38.6, 68.2)	37.0* (29.8, 44.7)	59.3 (44.4, 72.7)	33.5** (26.6, 41.2)
Prohibiting the possession of guns that do not have serial numbers	61.1 (54.0, 67.8)	68.1 (52.4, 80.6)	59.6 (51.6, 67.2)	62.7 (47.6, 75.6)	60.6 (52.4, 68.2)

Statistically significant difference indicated in bold

^*^p ≤ 0.05, **p ≤ 0.01, *** *p* ≤ 0.001

^1^Questions on purchase motivation were asked of all respondents who purchased a gun since January 1, 2020, including both new and prior gun owners. Recent purchases are defined here as purchases since January 1, 2020. Questions about motivation asked whether the recent purchases were motivated by concerns of racial violence and by concerns of political violence

^2^This question was only asked of those opposed to requiring a license from a local law enforcement agency before buying a gun to verify their identity and ensure that they are not legally prohibited from having a gun. All Recent Purchasers N = 145; Recent Purchase Motivated by Concerns of Racial Violence: Yes N = 23, No N = 122; Recent Purchase Motivated by Concerns of Political Violence: Yes N = 34, No N = 111

There were few differences between the unadjusted support and predicted probabilities of support that accounted for demographic characteristics, including political party affiliation (Supplemental Tables 3, 4 in Additional File 1). The predicted probabilities of support tended to be slightly lower than the unadjusted weighted proportions, but overall support and trends were similar.

## Discussion

New and prior gun owners differed in terms of purchase motivations and demographics, including political party affiliation, race, and ethnicity. Among those who purchased a gun since January 1, 2020, significantly larger proportions of Democrat, Black, Asian, and Hispanic respondents reported being new gun owners compared to Republican and white respondents. The demographics of new gun owners seen in this survey are consistent with prior literature, including results from the 2021 National Firearms Survey, which reported higher proportions of new gun ownership among Black and Hispanic adults, but our results add context on the motivations behind these shifts (Miller et al. 2022). Among recent purchasers, significantly larger proportions of Black and Asian respondents and new gun owners reported concerns of racial violence motivated purchases compared to white respondents and prior gun owners. Significantly larger proportions of Democrats and new gun owners reported concerns of political violence motivated recent purchases compared to Republicans and prior gun owners. Despite the differences in demographics between new and prior gun owners and between those who reported purchases motivated by concerns of racial or political violence and those who did not, the self-reported support for gun-related policies was largely similar between these groups, reflecting high support for protective policies across groups.

While the higher proportions of new gun ownership among Democrat, Black, Asian, and Hispanic respondents may be due in part to the fact that white individuals and Republicans had higher baseline rates of gun ownership before the pandemic, this still reflects a shift toward higher gun ownership rates among demographic groups that have historically had lower rates of gun ownership (Hill et al. 2021). If persistent, this shift may gradually lead to the demographics of gun owners in the U.S. becoming more representative of the population overall. Understanding this shifting demographic trend in gun ownership will help inform targeted messaging to groups regarding safe and responsible gun ownership.

Furthermore, understanding the factors that motivated pandemic-era gun purchases is essential to contextualizing the rise in new gun ownership. The odds of reporting purchases motivated by concerns of racial or political violence were 4.5- and 3.2-times higher, respectively, among new gun owners compared to prior gun owners who also made recent purchases. These purchase motivations among new gun owners are aligned with previously reported reasons for gun ownership measured in this survey, which found that 85% of new gun owners identified protection in ideological conflict as an important reason for gun ownership compared to only 56% of prior gun owners (Ward et al. 2024). Although personal interpretations of racial or political violence may vary (Kalyvas 2019), these concerns are not unfounded. Racially motivated hate crimes increased by 32% from 2019 to 2020, with the most significant increases in hate crimes targeting Black Americans (49% increase) and Asian Americans (77% increase) (U.S. Department of Justice 2023). Significantly larger proportions of both Black and Asian respondents reported concerns of racial violence motivating recent purchases compared to white respondents. The U.S. has also experienced a well-documented rise in support for and anticipation of violence to advance political objectives. A 2022 national survey found that 14% of Americans believe there will be civil war in the U.S. in the next few years and approximately one-third of Americans believe that political violence is usually or always justified to advance specific political objectives (Wintemute et al. 2023). Given such evidence of racial and political violence, increased gun purchasing and ownership, and the documented risk of increased lethality of violent situations when firearms are used, there is an urgent need for increased research and understanding of the drivers of these surges in violence and co-occurring harms (Braga et al. 2021). Opportunity remains for equitable and intentional collaboration between public health and public safety professionals to prevent such violence.

We examined recent purchases in the context of a gun purchasing surge coinciding with the emergence of COVID-19, but the concerns motivating this surge and driving the shifting demographics of new gun ownership are more complex and potentially more enduring than just the pandemic. These concerns of racial and political violence as motivators may be considered within the framework of the coping model of protective gun ownership, which posits that the motivation to own guns for protection is rooted in larger fears about the dangerousness of the world and that gun ownership helps individuals cope with psychological threats to their safety and security (Buttrick 2020). When examining motivations for recent purchases, stratification by race and ethnicity only revealed significant differences in the proportions of recent purchases motivated by racial violence; stratification by political party only revealed significant differences in the proportions motivated by political violence. Additionally, previously reported findings from this survey found that larger proportions of new gun owners endorsed protection during political activities (46%) and protection against people who do not share their beliefs (47%) as important reasons for gun ownership compared to prior gun owners (31% and 27%, respectively) (Ward et al. 2024). These results may anticipate trends in gun purchasing in response to future crises or times of social unrest, as they indicate that some individuals buy guns when they feel uncertain or unsafe; this may be especially true when individuals fear violence toward the political parties or racial or ethnic groups with which they identify. Given the efforts by the firearm industry to convincingly sell assurances of safety through guns, comparably strong public health messaging to promote safe and responsible firearm ownership and more comprehensive assurances of safety through clearly communicated and well implemented policy are needed (Hussain et al. 2023).

Despite notable shifts in the demographics and motivations of new gun owners, few differences in their self-reported support for different gun-related policies were observed. This lack of significant differences in policy support between new and prior gun owners is surprising given the documented differences in policy support by political party and race and ethnicity from past surveys, even among gun owners of differing political parties and races (Crifasi et al. 2021b; Burton et al. 2021). Still, as evidenced here and across past research, gun owners, including new gun owners, are broadly supportive of protective gun policies (Crifasi et al. 2021b; Barry et al. 2018). Additionally, both prior and new gun owners maintained strong support for protective gun policies, including requiring a background check system for all gun sales; prohibiting firearm ownership among those subject to a temporary domestic violence restraining order; prohibiting a person convicted of a serious crime as a juvenile form having a gun for 10 years; and requiring first-time purchasers to take a safety course on safe handling and storage before buying a gun. The only policy for which new and prior gun owners reported significantly different rates of support was requiring prospective gun purchasers to first get a license, with new gun owners expressing higher rates of support. This increased support for firearm purchaser licensing policies may be particularly important to interests in safer environments and communities, given the significant evidence supporting the efficacy of these policies in reducing gun homicide and suicide rates (Crifasi et al. 2015; Webster et al. 2014; McCourt et al. 2020).

Similarly, few differences in policy support were noted among recent purchasers who reported their purchase being motivated by concerns of racial or political violence and those who did not report such concerns, despite key demographic differences in those who reported such motivations. Notably, both those who reported recent purchase motivations of concerns of racial violence and of concerns of political violence reported greater levels of support for a stand-your-ground policy compared to those who did not report such motivations. This suggests a willingness to or acceptance of the ability to shoot and kill another individual when feeling threatened, potentially including these perceived threats of racial or political violence. This support for a stand-your-ground policy among those reporting that their firearm purchases were motivated by concerns of racial or political violence is concerning given the simultaneous rise in support for political violence and acceptance of use of force to achieve political aims (Pape 2023; Wintemute 2021). Still, as was the case among new and prior gun owners, overall support for protective policies was high regardless of purchase motivation. This suggests that, even as new groups turn to guns for personal protection, support for policies to promote safe and responsible ownership remains strong.

These findings should be considered in the context of various limitations. Sampling biases may have impacted our findings, but these biases are minimized through NORC at the University of Chicago’s probability-based sampling, which covers 95% or more of U.S. households (NORC at the University of Chicago 2022). Sample sizes for some of the groups that we analyzed, including new gun owners when broken down by race and ethnicity and political party, were small, limiting our capacity to examine other intersecting identities. Respondents identifying as non-Hispanic multiracial and non-Hispanic other race were grouped into one ethnoracial category for analysis, potentially masking differences in gun purchasing behaviors and policy preferences between and within these groups. We created dichotomous variables of policy support in our analysis, combining the neutral and opposition responses. This choice may have obscured information on policies with large proportions of neutral responses. Our findings may have also been impacted by social desirability bias and potential underreporting of firearm ownership (Bond et al. 2024), but these concerns are minimized by the use of an anonymous survey, and rates of personal gun ownership among respondents measured through this survey are often higher than other reports. Recall bias may have impacted our findings as well when asking respondents about their gun purchasing behaviors and motivations potentially up to three years after the purchase. However, asking about the most recent purchase, rather than all purchases or those further back in time, can help to mitigate this issue. The lack of explicit definitions of racial or political violence in the survey questions and the order of the survey questions may have led to response bias, but this bias was minimized by the randomization of question blocks. Recent purchasers were only asked to respond “Yes” or “No” regarding whether their recent purchase was motivated by concerns of racial or political violence, preventing examination of the strength or importance of these motivations. As with any survey, alternative question phrasing may yield different results. Future research may consider the use of more open-ended and varied questions to elicit a broader response regarding purchase motivations. Additionally, future studies may further examine whether purchasing motivations differ by gun type, as there is some evidence that pandemic-related concerns increased handgun, but not rifle, desirability (Sola 2021).

## Conclusions

Concerns of racial or political violence were cited as significant motivations for recent gun purchases among new gun owners, in particular among groups with historically lower rates of firearm ownership such as Democrats and Black, Asian, and Hispanic Americans. Understanding the demographics and motivations of new gun owners is essential to addressing concerns of targeted violence and crafting more effective messaging and interventions to encourage safe, responsible gun ownership. The belief that one’s only recourse for safety is to buy deadly weapons may lead to surges in firearm purchasing that further stoke, rather than resolve, the fear that motivated gun purchasing in the first place. Addressing concerns of racial or political violence, and the inequities in power and safety in American society that drive it, is a long-term challenge that public health must face.

## Supplementary Information

Additional file 1.

## Data Availability

The datasets generated and/or analyzed during the current study are not publicly available as analyses are ongoing, but the datasets will be made available to qualified researchers subject to the terms of a data use agreement.

## Abbreviation

CDC: Centers for Disease Control and Prevention
