# Supplementary material for: Political violence, racial violence, and new gun ownership: results from the 2023 National Survey of Gun Policy
Source: Inj Epidemiol. 2024 Sep 6;11:48. doi: 10.1186/s40621-024-00527-z (PMC11378614; doi:10.1186/s40621-024-00527-z)
Supplement: Supplementary file 1 — Additional file 1. [file 40621_2024_527_MOESM1_ESM.docx]

**Additional File 1: Supplemental Data Tables**

**Supplemental Table 1: Weighted and unweighted demographic characteristics of the study sample and national rates**

|  |  | **National Comparison**  **(%)** | **Unweighted**  **(%)** | **Weighted**  **(%)** |
| --- | --- | --- | --- | --- |
| Female | | 51.0 | 46.9 | 49.7 |
| Age | |  |  |  |
|  | 18-34 | 29.1 | 23.8 | 28.5 |
|  | 35-49 | 24.6 | 25.3 | 23.8 |
|  | 50-64 | 24.7 | 26.5 | 25.1 |
|  | 65+ | 21.6 | 24.5 | 22.6 |
| Race/Ethnicity | |  |  |  |
|  | White, non-Hispanic | 60.8 | 44.9 | 62.7 |
|  | Black, non-Hispanic | 11.7 | 21.6 | 12.0 |
|  | Asian, non-Hispanic | 6.1 | 10.9 | 6.3 |
|  | Other or multi-racial, non-Hispanic | 4.6 | 2.1 | 2.2 |
|  | Hispanic | 16.8 | 20.5 | 16.9 |
| Education | |  |  |  |
|  | High school diploma or less | 38.0 | 22.1 | 37.8 |
|  | Some college | 29.5 | 39.5 | 26.7 |
|  | Bachelor’s degree or higher | 32.4 | 38.4 | 35.5 |
| Household income | |  |  |  |
|  | <$35,000 | 25.2 | 28.1 | 28.8 |
|  | $35,000-$74,999 | 28.1 | 30.7 | 29.6 |
|  | >$75,000 | 46.8 | 41.2 | 41.6 |
| Employment status | |  |  |  |
|  | Employed | 61.3 | 60.5 | 58.7 |
|  | Unemployed | 2.3 | 6.3 | 6.8 |
|  | Other | 36.5 | 33.2 | 34.5 |
| Region | |  |  |  |
|  | Northeast | 17.6 | 13.0 | 17.2 |
|  | Midwest | 20.7 | 23.8 | 20.7 |
|  | South | 38.1 | 37.7 | 38.9 |
|  | West | 23.6 | 25.5 | 23.2 |
| Political Party Affiliation | |  |  |  |
|  | Republican | 31.5 | 23.6 | 28.5 |
|  | Independent | 33.8 | 37.6 | 38.6 |
|  | Democrat | 34.7 | 38.7 | 32.8 |

NOTE: National comparison data were obtained for the U.S. population ages 18+ from the American Community Survey (ACS) 1-year estimates for 2021, the 2020 Decennial Census, the 2022 Current Population Survey, and the American National Election Studies (ANES).

**Supplemental Table 2:** **Support for 42 different gun policies by gun ownership status**

|  |  | **Overall**  **% (CI)**  **(N=3096)** | **Gun owner**  **% (CI)**  **(N=1002)** | **Non-gun owner**  **% (CI)**  **(N=2094)** |
| --- | --- | --- | --- | --- |
| **License and background check policies** | |  |  |  |
|  | Requiring a background check system for all gun sales to make sure a purchaser is not legally prohibited from having a gun | 85.2  (83.3, 86.9) | 84.1  (81.0, 86.8) | 85.7  (83.3, 87.8) |
|  | Requiring a person to obtain a license from a local law enforcement agency before buying a gun to verify their identity and ensure that they are not legally prohibited from having a gun | 72.4  (70.1, 74.5) | 64.4  (60.5, 68.0) | **76.2*****  (73.4, 78.8) |
|  | Requiring a license to buy a gun if you could substitute a valid concealed carry license^1^ | 20.9  (17.1, 25.2) | 20.9  (16.4, 26.3) | 20.9  (15.5, 27.4) |
|  | Requiring that a person be fingerprinted for the background check to verify a person’s identity and link it to any relevant criminal records | 78.3  (76.2, 80.3) | 71.2  (67.4, 74.6) | **81.7*****  (79.1, 84.0) |
|  | Extending the time to conduct a background check to up to 10 days | 68.8  (66.5, 71.0) | 62.6  (58.7, 66.3) | **71.7*****  (68.8, 74.4) |
|  | Prohibiting the sale of a gun before a background check is complete | 65.7  (63.3, 68.0) | 65.1  (61.3, 68.8) | 66.0  (63.0, 68.9) |
| **Prohibited persons policies** | |  |  |  |
|  | Prohibiting a person subject to a temporary domestic violence restraining order from having a gun for the duration of the order | 80.9  (78.9, 82.8) | 79.2  (75.8, 82.3) | 81.7  (79.1, 84.0) |
|  | Extending domestic violence-related gun prohibitions to include couples who have dated | 61.6  (59.2, 64.0) | 57.1  (53.2, 61.0) | **63.7****  (60.7, 66.7) |
|  | Prohibiting a person convicted of a serious crime as a juvenile from having a gun for 10 years | 77.4  (75.3, 79.4) | 77.8  (74.5, 80.8) | 77.2  (74.5, 79.7) |
|  | Prohibiting a person under the age of 21 from having a handgun | 67.0  (64.6, 69.3) | 57.7  (53.7, 61.5) | **71.4*****  (68.5, 74.2) |
|  | Prohibiting a person convicted of two or more misdemeanor crimes involving illegal drugs in a five-year period from having a gun for five years | 64.3  (61.9, 66.6) | 61.0  (57.1, 64.8) | **65.8***  (62.8, 68.7) |
|  | Prohibiting a person convicted of two or more DWI or DUIs in a five-year period from having a gun for five years | 59.4  (56.9, 61.7) | 52.7  (48.8, 56.6) | **62.5*****  (59.5, 65.5) |
| **Assault weapon and ammunition policies** | |  |  |  |
|  | Banning the sale of military-style, semi-automatic assault weapons that are capable of shooting more than 10 rounds of ammunition without reloading | 59.4  (57.0, 61.8) | 44.2  (40.4, 48.2) | **66.6*****  (63.6, 69.5) |
|  | Banning the sale of large-capacity ammunition clips or magazines that allow some guns to shoot more than 10 bullets before reloading | 58.0  (55.6, 60.4) | 43.4  (39.5, 47.3) | **65.0*****  (61.9, 67.9) |
|  | Requiring an owner of a semi-automatic rifle, that ejects and rechambers a new round after each shot allowing a person to fire the rifle as quickly as the trigger can be pulled, to be at least 21 years of age | 72.5  (70.3, 74.5) | 65.9  (62.1, 69.4) | **75.6*****  (73.0, 78.1) |
| **Policies affecting gun dealers** | |  |  |  |
|  | Allowing the Bureau of Alcohol, Tobacco and Firearms to temporarily take away a gun dealer’s license if an audit reveals record-keeping violations and the dealer cannot account for 20 or more of his guns | 80.2  (78.1, 82.2) | 80.2  (76.7, 83.2) | 80.2  (77.5, 82.7) |
|  | Allowing cities to sue licensed gun dealers when there is strong evidence that the gun dealer’s careless sales practices allowed many criminals to obtain guns | 71.7  (69.4, 73.8) | 68.6  (64.9, 72.2) | 73.1  (70.3, 75.8) |
|  | Allowing the information about which gun dealers sell the most guns used in crimes to be available to the police and the public so that those gun dealers can be prioritized for greater oversight | 64.9  (62.5, 67.2) | 61.1  (57.2, 64.9) | **66.7***  (63.6, 69.6) |
| **Temporary firearm removal policies** | |  |  |  |
|  | Allowing family members to ask the court to temporarily remove guns from a relative who they believe is at risk of harming himself or others | 76.4  (74.2, 78.4) | 72.3  (68.6, 75.7) | **78.4****  (75.7, 80.8) |
|  | Authorizing law enforcement officers to temporarily remove guns from individuals who the officer determines pose an immediate threat of harm to self or others | 71.3  (69.0, 73.4) | 65.5  (61.7, 69.2) | **74.0*****  (71.2, 76.6) |
|  | Allowing licensed healthcare providers to ask the court to temporarily remove guns from a patient who they believe is at risk of harming himself or others | 76.4  (74.2, 78.4) | 71.6  (67.8, 75.0) | **78.7*****  (76.0, 81.1) |
| **Policies on carrying guns in public** | |  |  |  |
|  | Requiring a person who has applied for a license to carry a concealed gun in public to pass a test demonstrating that they can safely and lawfully handle a gun in common situations they might encounter | 72.7  (70.4, 74.9) | 68.3  (64.5, 71.9) | **74.8****  (72.0, 77.4) |
|  | Allowing a person who can legally carry a concealed gun to bring that gun onto a college or university campus | 27.3  (25.2, 29.4) | 41.8  (38.0, 45.8) | **20.3*****  (18.0, 22.9) |
|  | Allowing a person who can legally carry a concealed gun to bring that gun onto school grounds for kindergarten through 12th grade | 25.2  (23.2, 27.4) | 36.3  (32.6, 40.2) | **20.0*****  (17.6, 22.7) |
|  | Allowing a person who can legally own a gun to carry a loaded, concealed handgun in public without having to obtain a concealed carry license | 22.8  (20.9, 25.0) | 34.5  (30.9, 38.2) | **17.3*****  (15.0, 19.9) |
|  | Requiring a state to recognize a concealed carry permit from another state, even if that other state’s firearm concealed carry permitting standards are lower | 47.4  (45.0, 49.9) | 62.7  (58.7, 66.4) | **40.1*****  (37.1, 43.2) |
|  | Prohibiting the open carrying of a gun (i.e., carrying in a manner that makes it visible) at a public demonstration or rally | 56.8  (54.3, 59.2) | 48.0  (44.1, 51.9) | **60.9*****  (57.9, 63.9) |
|  | Prohibiting a person from bringing a gun into a government building | 68.1  (65.8, 70.3) | 59.4  (55.5, 63.2) | **72.2*****  (69.4, 74.9) |
| **Policies prohibiting a person convicted of each of these crimes from having a gun for 10 years** | |  |  |  |
|  | Public display of a gun in a threatening manner, excluding self-defense | 69.6  (67.3, 71.8) | 69.8  (66.0, 73.3) | 69.5  (66.6, 72.3) |
|  | Assault and battery that does not result in serious injury or involve a lethal weapon | 53.6  (51.2, 56.0) | 49.8  (45.9, 53.7) | **55.5***  (52.4, 58.5) |
|  | Carrying a concealed gun without a permit | 52.2  (49.7, 54.6) | 46.4  (42.5, 50.3) | **54.9*****  (51.8, 58.0) |
|  | Drunk and disorderly conduct | 44.9  (42.4, 47.3) | 37.7  (34.0, 41.6) | **48.3*****  (45.2, 51.4) |
| **Funding-related policies** | |  |  |  |
|  | Directing federal government funding to states that want to establish licensing systems for handgun purchasers | 55.7  (53.3, 58.1) | 49.8  (45.8, 53.7) | **58.6*****  (55.5, 61.6) |
|  | Funding community-based gun violence prevention programs that provide outreach, conflict mediation, and social support for individuals at high risk of gun violence | 69.0  (66.7, 71.3) | 61.8  (57.8, 65.6) | **72.5*****  (69.6, 75.2) |
|  | Directing public funding to dispatching a clinician to accompany police officers on calls involving individuals displaying symptoms of mental illness | 67.8  (65.5, 70.0) | 62.6  (58.7, 66.4) | **70.2****  (67.4, 73.0) |
|  | Directing public funding for community-based mental health programs to respond to calls involving individuals displaying symptoms of mental illness | 71.4  (69.1, 73.6) | 65.8  (61.9, 69.5) | **74.1*****  (71.3, 76.7) |
|  | Redirecting government funding currently spend on the police to social services for people at risk of gun violence | 38.6  (36.3, 41.0) | 29.3  (25.9, 33.0) | **43.1*****  (40.1, 46.2) |
|  | Funding, through public insurance, hospital-based gun violence prevention programs that offer counseling to address psychological trauma | 61.9  (59.5, 64.2) | 54.0  (50.1, 58.0) | **65.6*****  (62.6, 68.4) |
| **Safe storage policies** | |  |  |  |
|  | Requiring first-time gun purchasers to take a safety course on safe handling and storage before buying a gun | 83.1  (81.2, 85.0) | 78.8  (75.4, 81.8) | **85.2*****  (82.7, 87.4) |
|  | Requiring by law that a person lock up the guns in their home when not in use to prevent handling by children or teenagers without adult supervision | 72.2  (70.0, 74.3) | 58.0  (54.1, 61.9) | **78.9*****  (76.3, 81.3) |
| **Other policies** | |  |  |  |
|  | Allowing a person with a gun who feels a threat of serious injury from another person to shoot or kill that threatening person, even if the gun owner could safely retreat | 27.1  (25.0, 29.3) | 35.9  (32.2, 39.7) | **22.9*****  (20.3, 25.7) |
|  | Prohibiting the possession of guns that do not have serial numbers | 72.8  (70.5, 75.0) | 66.2  (62.3, 69.8) | **76.0*****  (73.2, 78.7) |

Statistically significant difference indicated in bold: *p≤0.05, **p≤0.01, *** p≤0.001

^1^This question was only asked of those opposed to requiring a license from a local law enforcement agency before buying a gun to verify their identity and ensure that they are not legally prohibited from having a gun. Overall N=787, Gun Owner N=352, Non-Gun Owner N=435

**Supplemental Table 3: Predicted probabilities of gun policy support by new versus prior gun ownership**

|  |  | **Overall Gun Owner**  **% (CI)**  **(N=1002)** | **Prior Gun Owner**  **% (CI)**  **(N=904)** | **New Gun Owner**  **% (CI)**  **(N=119)** |
| --- | --- | --- | --- | --- |
| **License and background check policies** | |  |  |  |
|  | Requiring a background check system for all gun sales to make sure a purchaser is not legally prohibited from having a gun | 84.7  (81.8, 87.6) | 83.6  (80.7, 86.6) | 87.2  (79.3, 95.2) |
|  | Requiring a person to obtain a license from a local law enforcement agency before buying a gun to verify their identity and ensure that they are not legally prohibited from having a gun | 67.3  (63.6, 70.9) | 63.2  (59.5, 67.0) | **75.7***  **(65.2, 86.1)** |
|  | Requiring a license to buy a gun if you could substitute a valid concealed carry license^1^ | 21.9  (16.3, 27.4) | 19.3  (14.4, 24.2) | 38.3  (16.6, 60.0) |
|  | Requiring that a person be fingerprinted for the background check to verify a person’s identity and link it to any relevant criminal records | 73.2  (69.7, 76.7) | 69.9  (66.3, 73.5) | 81.8  (72.1, 91.5) |
|  | Extending the time to conduct a background check to up to 10 days | 63.7  (59.7, 67.7) | 62.3  (58.5, 66.1) | 66.1  (54.4, 77.7) |
|  | Prohibiting the sale of a gun before a background check is complete | 65.4  (61.3, 69.4) | 65.0  (61.1, 68.9) | 63.0  (50.8, 75.1) |
| **Prohibited persons policies** | |  |  |  |
|  | Prohibiting a person subject to a temporary domestic violence restraining order from having a gun for the duration of the order | 80.4  (77.2, 83.5) | 79.0  (75.7, 82.2) | 79.8  (70.1, 89.4) |
|  | Extending domestic violence-related gun prohibitions to include couples who have dated | 59.9  (55.9, 63.8) | 57.1  (53.2, 61.0) | 56.7  (44.7, 68.7) |
|  | Prohibiting a person convicted of a serious crime as a juvenile from having a gun for 10 years | 78.0  (74.8, 81.2) | 77.4  (74.2, 80.7) | 79.9  (70.6, 89.2) |
|  | Prohibiting a person under the age of 21 from having a handgun | 62.5  (58.7, 66.3) | 57.3  (53.5, 61.1) | 64.9  (53.0, 76.8) |
|  | Prohibiting a person convicted of two or more misdemeanor crimes involving illegal drugs in a five-year period from having a gun for five years | 62.5  (58.6, 66.4) | 61.1  (57.2, 64.9) | 62.6  (51.6, 73.5) |
|  | Prohibiting a person convicted of two or more DWI or DUIs in a five-year period from having a gun for five years | 56.3  (52.4, 60.2) | 52.8  (49.0, 56.7) | 56.2  (45.0, 67.5) |
| **Assault weapon and ammunition policies** | |  |  |  |
|  | Banning the sale of military-style, semi-automatic assault weapons that are capable of shooting more than 10 rounds of ammunition without reloading | 49.4  (45.5, 53.3) | 44.6  (40.9, 48.2) | 41.2  (29.6, 52.7) |
|  | Banning the sale of large-capacity ammunition clips or magazines that allow some guns to shoot more than 10 bullets before reloading | 47.0  (42.9, 51.0) | 43.6  (39.8, 47.4) | 41.5  (30.1, 52.9) |
|  | Requiring an owner of a semi-automatic rifle, that ejects and rechambers a new round after each shot allowing a person to fire the rifle as quickly as the trigger can be pulled, to be at least 21 years of age | 67.9  (64.2, 71.5) | 65.4  (61.8, 69.0) | 71.8  (61.6, 82.1) |
| **Policies affecting gun dealers** | |  |  |  |
|  | Allowing the Bureau of Alcohol, Tobacco and Firearms to temporarily take away a gun dealer’s license if an audit reveals record-keeping violations and the dealer cannot account for 20 or more of his guns | 80.6  (77.3, 83.9) | 80.5  (77.3, 83.8) | 76.6  (65.7, 87.4) |
|  | Allowing cities to sue licensed gun dealers when there is strong evidence that the gun dealer’s careless sales practices allowed many criminals to obtain guns | 70.9  (67.3, 74.5) | 68.6  (64.9, 72.2) | 67.2  (55.4, 79.0) |
|  | Allowing the information about which gun dealers sell the most guns used in crimes to be available to the police and the public so that those gun dealers can be prioritized for greater oversight | 63.2  (59.3, 67.2) | 61.1  (57.2, 65.0) | 58.6  (46.7, 70.6) |
| **Temporary firearm removal policies** | |  |  |  |
|  | Allowing family members to ask the court to temporarily remove guns from a relative who they believe is at risk of harming himself or others | 74.5  (71.0, 78.0) | 72.4  (68.8, 76.0) | 68.4  (57.0, 79.8) |
|  | Authorizing law enforcement officers to temporarily remove guns from individuals who the officer determines pose an immediate threat of harm to self or others | 67.0  (63.2, 70.8) | 65.4  (61.6, 69.2) | 64.0  (52.7, 75.3) |
|  | Allowing licensed healthcare providers to ask the court to temporarily remove guns from a patient who they believe is at risk of harming himself or others | 74.5  (71.0, 77.9) | 71.3  (67.7, 75.0) | 70.8  (60.5, 81.2) |
| **Policies on carrying guns in public** | |  |  |  |
|  | Requiring a person who has applied for a license to carry a concealed gun in public to pass a test demonstrating that they can safely and lawfully handle a gun in common situations they might encounter | 68.4  (64.5, 72.3) | 67.8  (64.0, 71.6) | 72.3  (61.9, 82.6) |
|  | Allowing a person who can legally carry a concealed gun to bring that gun onto a college or university campus | 36.8  (32.9, 40.6) | 41.4  (37.5, 45.2) | 40.3  (28.3, 52.4) |
|  | Allowing a person who can legally carry a concealed gun to bring that gun onto school grounds for kindergarten through 12th grade | 32.6  (28.9, 36.3) | 35.6  (31.9, 39.3) | 42.9  (31.1, 54.6) |
|  | Allowing a person who can legally own a gun to carry a loaded, concealed handgun in public without having to obtain a concealed carry license | 31.7  (28.0, 35.4) | 33.8  (30.1, 37.5) | 39.3  (27.4, 51.1) |
|  | Requiring a state to recognize a concealed carry permit from another state, even if that other state’s firearm concealed carry permitting standards are lower | 61.5  (57.4, 65.6) | 61.4  (57.5, 65.3) | 70.3  (60.2, 80.3) |
|  | Prohibiting the open carrying of a gun (i.e., carrying in a manner that makes it visible) at a public demonstration or rally | 49.7  (45.5, 53.8) | 47.5  (43.5, 51.5) | 53.0  (40.9, 65.0) |
|  | Prohibiting a person from bringing a gun into a government building | 61.9  (58.0, 65.8) | 58.8  (55.0, 62.7) | 68.1  (57.9, 78.3) |
| **Policies prohibiting a person convicted of each of these crimes from having a gun for 10 years** | |  |  |  |
|  | Public display of a gun in a threatening manner, excluding self-defense | 69.3  (65.4, 73.3) | 69.9  (66.1, 73.7) | 70.4  (59.4, 81.4) |
|  | Assault and battery that does not result in serious injury or involve a lethal weapon | 51.0  (46.8, 55.2) | 50.2  (46.2, 54.2) | 50.2  (37.7, 62.6) |
|  | Carrying a concealed gun without a permit | 47.7  (43.7, 51.8) | 45.7  (41.8, 49.6) | 53.6  (42.0, 65.1) |
|  | Drunk and disorderly conduct | 39.9  (35.8, 44.0) | 37.1  (33.3, 41.0) | 45.9  (33.7, 58.2) |
| **Funding-related policies** | |  |  |  |
|  | Directing federal government funding to states that want to establish licensing systems for handgun purchasers | 51.3  (47.3, 55.4) | 49.7  (45.8, 53.7) | 48.7  (35.2, 62.3) |
|  | Funding community-based gun violence prevention programs that provide outreach, conflict mediation, and social support for individuals at high risk of gun violence | 64.7  (60.8, 68.6) | 61.8  (58.1, 65.5) | 62.2  (50.3, 74.0) |
|  | Directing public funding to dispatching a clinician to accompany police officers on calls involving individuals displaying symptoms of mental illness | 65.7  (61.7, 69.6) | 62.7  (58.9, 66.6) | 59.0  (46.5, 71.6) |
|  | Directing public funding for community-based mental health programs to respond to calls involving individuals displaying symptoms of mental illness | 67.6  (63.8, 71.4) | 65.4  (61.6, 69.2) | 66.5  (54.0, 78.9) |
|  | Redirecting government funding currently spend on the police to social services for people at risk of gun violence | 34.1  (30.3, 37.9) | 29.7  (26.3, 33.1) | 25.0  (15.7, 34.2) |
|  | Funding, through public insurance, hospital-based gun violence prevention programs that offer counseling to address psychological trauma | 58.0  (54.0, 62.0) | 53.7  (49.7, 57.6) | 52.8  (39.8, 65.9) |
| **Safe storage policies** | |  |  |  |
|  | Requiring first-time gun purchasers to take a safety course on safe handling and storage before buying a gun | 79.7  (76.3, 83.0) | 78.2  (74.9, 81.6) | 81.1  (71.2, 90.9) |
|  | Requiring by law that a person lock up the guns in their home when not in use to prevent handling by children or teenagers without adult supervision | 62.6  (58.7, 66.4) | 57.2  (53.3, 61.1) | 64.7  (52.7, 76.7) |
| **Other policies** | |  |  |  |
|  | Allowing a person with a gun who feels a threat of serious injury from another person to shoot or kill that threatening person, even if the gun owner could safely retreat | 33.5  (29.7, 37.3) | 35.8  (32.0, 39.6) | 33.6  (22.3, 44.8) |
|  | Prohibiting the possession of guns that do not have serial numbers | 67.8  (63.9, 71.7) | 65.7  (61.9, 69.5) | 69.8  (57.9, 81.6) |

Statistically significant difference indicated in bold: *p≤0.05, **p≤0.01, *** p≤0.001

NOTE: Predicted probabilities were calculated while controlling for political party, race and ethnicity, sex, age, income, and urbanicity.

^1^This question was only asked of those opposed to requiring a license from a local law enforcement agency before buying a gun to verify their identity and ensure that they are not legally prohibited from having a gun. Overall Gun Owner N=352, Prior Gun Owner N=325, New Gun Owner N=32

**Supplemental Table 4: Predicted probabilities of gun policy support by motivation for recent gun purchases**

|  |  |  | **Recent Purchase Motivated by Concerns of Racial Violence^1^** | | **Recent Purchase Motivated by Concerns of Political Violence^1^** | |
| --- | --- | --- | --- | --- | --- | --- |
|  |  | **All Recent Purchases**  **% (CI)**  **(N=327)** | **Yes**  **% (CI)**  **(N=69)** | **No**  **% (CI)**  **(N=258)** | **Yes**  **% (CI)**  **(N=83)** | **No**  **% (CI)**  **(N=244)** |
| **License and background check policies** | |  |  |  |  |  |
|  | Requiring a background check system for all gun sales to make sure a purchaser is not legally prohibited from having a gun | 82.3  (77.1, 87.5) | 81.8  (70.3, 93.2) | 78.8  (72.9, 84.7) | 81.0  (70.7, 91.4) | 78.7  (72.5, 85.0) |
|  | Requiring a person to obtain a license from a local law enforcement agency before buying a gun to verify their identity and ensure that they are not legally prohibited from having a gun | 62.1  (55.5, 68.6) | 60.6  (45.5, 75.7) | 54.8  (47.6, 62.0) | 59.9  (47.0, 72.8) | 54.5  (46.9, 62.0) |
|  | Requiring a license to buy a gun if you could substitute a valid concealed carry license^2^ | 17.5  (10.5, 24.6) | 44.4  (17.9, 70.9) | **14.0***  **(7.6, 20.4)** | 36.1  (16.9, 55.4) | **13.2***  **(6.5, 19.9)** |
|  | Requiring that a person be fingerprinted for the background check to verify a person’s identity and link it to any relevant criminal records | 68.2  (61.9, 74.5) | 63.3  (46.1, 80.5) | 61.6  (54.8, 68.3) | 66.0  (51.7, 80.3) | 60.5  (53.4, 67.7) |
|  | Extending the time to conduct a background check to up to 10 days | 55.8  (48.8, 62.9) | 52.3  (35.6, 69.1) | 52.6  (45.4, 59.9) | 56.1  (42.3, 69.9) | 51.5  (43.9, 59.1) |
|  | Prohibiting the sale of a gun before a background check is complete | 59.7  (52.5, 66.9) | 55.9  (39.9, 72.0) | 59.3  (52.3, 66.3) | 66.8  (54.2, 79.3) | 56.3  (48.9, 63.7) |
| **Prohibited persons policies** | |  |  |  |  |  |
|  | Prohibiting a person subject to a temporary domestic violence restraining order from having a gun for the duration of the order | 77.8  (72.2, 83.3) | 72.4  (58.1, 86.8) | 73.3  (66.5, 80.0) | 71.0  (58.4, 83.5) | 73.8  (66.8, 80.8) |
|  | Extending domestic violence-related gun prohibitions to include couples who have dated | 57.2  (50.2, 64.1) | 47.2  (32.0, 62.4) | 51.4  (43.8, 59.0) | 45.8  (31.8, 59.8) | 52.3  (44.4, 60.1) |
|  | Prohibiting a person convicted of a serious crime as a juvenile from having a gun for 10 years | 77.6  (72.2, 83.1) | 74.4  (61.5, 87.4) | 74.2  (67.9, 80.4) | 71.2  (59.2, 83.3) | 75.1  (68.7, 81.5) |
|  | Prohibiting a person under the age of 21 from having a handgun | 60.7  (54.0, 67.5) | 61.5  (44.5, 78.5) | 51.0  (43.7, 58.4) | 50.2  (35.7, 64.8) | 53.4  (45.9, 61.0) |
|  | Prohibiting a person convicted of two or more misdemeanor crimes involving illegal drugs in a five-year period from having a gun for five years | 58.6  (51.5, 65.7) | 64.7  (49.6, 79.8) | 51.6  (44.2, 59.1) | 61.7  (48.3, 75.0) | 51.4  (43.6, 59.1) |
|  | Prohibiting a person convicted of two or more DWI or DUIs in a five-year period from having a gun for five years | 56.1  (49.3, 63.0) | 56.2  (39.5, 72.9) | 48.8  (41.6, 56.0) | 60.3  (47.0, 73.6) | 46.9  (39.3, 54.4) |
| **Assault weapon and ammunition policies** | |  |  |  |  |  |
|  | Banning the sale of military-style, semi-automatic assault weapons that are capable of shooting more than 10 rounds of ammunition without reloading | 37.5  (30.6, 44.5) | 29.2  (15.6, 42.9) | 27.5  (20.8, 34.2) | 36.2  (22.3, 50.0) | 25.0  (18.7, 31.2) |
|  | Banning the sale of large-capacity ammunition clips or magazines that allow some guns to shoot more than 10 bullets before reloading | 34.7  (27.6, 41.9) | 31.3  (18.3, 44.3) | 25.6  (18.9, 32.2) | 30.6  (19.8, 41.5) | 25.2  (18.1, 32.3) |
|  | Requiring an owner of a semi-automatic rifle, that ejects and rechambers a new round after each shot allowing a person to fire the rifle as quickly as the trigger can be pulled, to be at least 21 years of age | 63.9  (57.4, 70.4) | 68.4  (53.4, 83.5) | 55.8  (48.3, 63.3) | 65.0  (50.2, 79.7) | 55.7  (48.1, 63.3) |
| **Policies affecting gun dealers** | |  |  |  |  |  |
|  | Allowing the Bureau of Alcohol, Tobacco and Firearms to temporarily take away a gun dealer’s license if an audit reveals record-keeping violations and the dealer cannot account for 20 or more of his guns | 77.9  (72.3, 83.6) | 81.2  (69.4, 93.1) | 72.1  (65.3, 78.8) | 73.0  (60.0, 86.0) | 73.8  (67.0, 80.6) |
|  | Allowing cities to sue licensed gun dealers when there is strong evidence that the gun dealer’s careless sales practices allowed many criminals to obtain guns | 67.5  (61.0, 74.0) | 63.7  (49.1, 78.4) | 60.7  (53.6, 67.8) | 66.7  (54.5, 79.0) | 59.4  (52.0, 66.9) |
|  | Allowing the information about which gun dealers sell the most guns used in crimes to be available to the police and the public so that those gun dealers can be prioritized for greater oversight | 54.6  (47.3, 61.8) | 52.1  (36.2, 68.0) | 46.3  (38.7, 53.9) | 53.0  (37.8, 68.2) | 45.4  (37.7, 53.2) |
| **Temporary firearm removal policies** | |  |  |  |  |  |
|  | Allowing family members to ask the court to temporarily remove guns from a relative who they believe is at risk of harming himself or others | 69.8  (63.6, 76.1) | 70.0  (55.8, 84.2) | 60.9  (53.5, 68.3) | 67.2  (53.9, 80.5) | 60.8  (53.2, 68.5) |
|  | Authorizing law enforcement officers to temporarily remove guns from individuals who the officer determines pose an immediate threat of harm to self or others | 57.4  (50.5, 64.3) | 48.1  (33.4, 62.8) | 50.1  (42.7, 57.5) | 47.4  (33.2, 61.6) | 50.5  (43.1, 58.0) |
|  | Allowing licensed healthcare providers to ask the court to temporarily remove guns from a patient who they believe is at risk of harming himself or others | 71.1  (65.1, 77.0) | 72.3  (58.5, 86.2) | 61.6  (54.4, 68.8) | 73.0  (60.8, 85.2) | 60.3  (52.8, 67.8) |
| **Policies on carrying guns in public** | |  |  |  |  |  |
|  | Requiring a person who has applied for a license to carry a concealed gun in public to pass a test demonstrating that they can safely and lawfully handle a gun in common situations they might encounter | 67.8  (61.4, 74.3) | 73.2  (59.3, 87.2) | 63.1  (56.0, 70.2) | 77.6  (67.1, 88.1) | **60.8***  **(53.3, 68.4)** |
|  | Allowing a person who can legally carry a concealed gun to bring that gun onto a college or university campus | 42.2  (35.4, 49.0) | 43.2  (26.2, 60.2) | 52.2  (45.0, 59.3) | 62.0  (48.6, 75.3) | 46.9  (39.3, 54.5) |
|  | Allowing a person who can legally carry a concealed gun to bring that gun onto school grounds for kindergarten through 12th grade | 36.5  (30.0, 43.0) | 43.3  (27.7, 58.9) | 43.5  (36.6, 50.4) | 47.0  (33.3, 60.8) | 42.3  (35.2, 49.4) |
|  | Allowing a person who can legally own a gun to carry a loaded, concealed handgun in public without having to obtain a concealed carry license | 35.8  (29.4, 42.3) | 58.1  (42.9, 73.3) | **39.0***  **(31.9, 46.2)** | 51.7  (38.4, 65.0) | 39.3  (31.8, 46.7) |
|  | Requiring a state to recognize a concealed carry permit from another state, even if that other state’s firearm concealed carry permitting standards are lower | 65.5  (58.3, 72.7) | 83.0  (73.1, 92.9) | **65.8***  **(58.2, 73.3)** | 79.0  (67.1, 90.9) | 65.4  (57.8, 73.0) |
|  | Prohibiting the open carrying of a gun at a public demonstration or rally? By open carry, we mean carrying a gun in a manner that makes it visible | 41.5  (34.0, 49.1) | 43.4  (27.5, 59.4) | 34.3  (26.9, 41.7) | 42.0  (28.9, 55.1) | 33.9  (26.2, 41.6) |
|  | Prohibiting a person from bringing a gun into a government building | 60.2  (53.6, 66.9) | 54.5  (39.2, 69.9) | 52.3  (45.0, 59.6) | 58.3  (44.9, 71.7) | 50.9  (43.2, 58.6) |
| **Policies prohibiting a person convicted of each of these crimes from having a gun for 10 years** | |  |  |  |  |  |
|  | Public display of a gun in a threatening manner, excluding self-defense | 69.1  (62.5, 75.7) | 71.0  (57.5, 84.4) | 66.7  (59.4, 74.0) | 62.3  (48.7, 75.9) | 69.0  (61.7, 76.2) |
|  | Assault and battery that does not result in serious injury or involve a lethal weapon | 49.1  (41.7, 56.5) | 50.8  (34.7, 66.9) | 44.9  (37.2, 52.6) | 46.1  (31.7, 60.5) | 45.9  (38.0, 53.8) |
|  | Carrying a concealed gun without a permit | 48.4  (41.2, 55.5) | 49.6  (34.4, 64.9) | 41.7  (34.2, 49.3) | 47.3  (32.5, 62.1) | 41.7  (34.0, 49.3) |
|  | Drunk and disorderly conduct | 38.3  (31.1, 45.5) | 37.7  (22.9, 52.4) | 33.1  (25.7, 40.5) | 38.6  (24.8, 52.5) | 32.3  (25.0, 39.6) |
| **Funding-related policies** | |  |  |  |  |  |
|  | Directing federal government funding to states that want to establish licensing systems for handgun purchasers | 46.8  (39.3, 54.4) | 51.0  (34.7, 67.3) | 39.7  (32.0, 47.4) | 43.7  (28.9, 58.4) | 40.9  (33.0, 48.7) |
|  | Funding community-based gun violence prevention programs that provide outreach, conflict mediation, and social support for individuals at high risk of gun violence | 59.8  (53.0, 66.7) | 53.2  (38.5, 67.8) | 53.5  (46.1, 60.9) | 52.0  (38.2, 65.7) | 54.0  (46.3, 61.6) |
|  | Directing public funding to dispatching a clinician to accompany police officers on calls involving individuals displaying symptoms of mental illness | 59.2  (52.1, 66.3) | 55.1  (39.5, 70.7) | 53.4  (46.2, 60.7) | 64.7  (51.8, 77.6) | 50.3  (42.7, 57.9) |
|  | Directing public funding for community-based mental health programs to respond to calls involving individuals displaying symptoms of mental illness | 61.0  (54.0, 68.1) | 66.5  (51.0, 81.9) | 54.5  (47.1, 61.9) | 64.6  (50.3, 78.9) | 53.9  (46.4, 61.5) |
|  | Redirecting government funding currently spend on the police to social services for people at risk of gun violence | 27.9  (21.6, 34.2) | 33.1  (20.2, 46.1) | 21.4  (15.8, 26.9) | 30.5  (19.3, 41.7) | 21.0  (15.2, 26.9) |
|  | Funding, through public insurance, hospital-based gun violence prevention programs that offer counseling to address psychological trauma | 52.9  (45.4, 60.4) | 52.2  (35.8, 68.7) | 46.0  (38.4, 53.5) | 50.2  (36.3, 64.1) | 46.0  (38.2, 53.8) |
| **Safe storage policies** | |  |  |  |  |  |
|  | Requiring first-time gun purchasers to take a safety course on safe handling and storage before buying a gun | 78.3  (72.8, 83.8) | 73.7  (61.0, 86.4) | 74.2  (68.0, 80.5) | 74.4  (63.3, 85.5) | 74.1  (67.4, 80.7) |
|  | Requiring by law that a person lock up the guns in their home when not in use to prevent handling by children or teenagers without adult supervision | 61.7  (54.8, 68.5) | 59.5  (41.9, 77.1) | 51.5  (44.3, 58.7) | 66.7  (53.8, 79.6) | **48.6***  **(41.1, 56.1)** |
| **Other policies** | |  |  |  |  |  |
|  | Allowing a person with a gun who feels a threat of serious injury from another person to shoot or kill that threatening person, even if the gun owner could safely retreat | 34.1  (27.7, 40.5) | 53.6  (39.2, 68.0) | **37.1***  **(30.0, 44.2)** | 58.8  (45.9, 71.7) | **33.8*****  **(26.8, 40.8)** |
|  | Prohibiting the possession of guns that do not have serial numbers | 67.1  (60.5, 73.6) | 62.5  (46.4, 78.5) | 60.8  (53.5, 68.2) | 61.0  (47.1, 74.9) | 61.1  (53.5, 68.7) |

Statistically significant difference indicated in bold: *p≤0.05, **p≤0.01, *** p≤0.001

NOTE: Predicted probabilities were calculated while controlling for political party, race and ethnicity, sex, age, income, and urbanicity.

^1^Questions on purchase motivation were asked of all respondents who purchased a gun since January 1, 2020, including both new and prior gun owners. Recent purchases are defined here as purchases since January 1, 2020. Questions about motivation asked whether the recent purchases were motivated by concerns of racial violence and by concerns of political violence.

^2^This question was only asked of those opposed to requiring a license from a local law enforcement agency before buying a gun to verify their identity and ensure that they are not legally prohibited from having a gun. All Recent Purchasers N=145; Recent Purchase Motivated by Concerns of Racial Violence: Yes N=23, No N=122; Recent Purchase Motivated by Concerns of Political Violence: Yes N=34, No N=111
